# Housework or vigilance? Bilbies alter their burrowing activity under threat of predation by feral cats

**DOI:** 10.1093/beheco/arad073

**Published:** 2023-10-31

**Authors:** Faith S E Chen, Stuart J Dawson, Patricia A Fleming

**Affiliations:** Terrestrial Ecosystem Science and Sustainability, Harry Butler Institute, Murdoch University, 90 South Street, Murdoch, Perth, Western Australia 6150, Australia; Terrestrial Ecosystem Science and Sustainability, Harry Butler Institute, Murdoch University, 90 South Street, Murdoch, Perth, Western Australia 6150, Australia; Terrestrial Ecosystem Science and Sustainability, Harry Butler Institute, Murdoch University, 90 South Street, Murdoch, Perth, Western Australia 6150, Australia

**Keywords:** biopedturbation, digging mammal, invasive species, landscape of fear, non-consumptive predator effect, predation risk allocation hypothesis

## Abstract

Behavioral adjustments to predation risk not only impose costs on prey species themselves but can also have cascading impacts on whole ecosystems. The greater bilby (*Macrotis lagotis*) is an important ecosystem engineer, modifying the physical environment through their digging activity, and supporting a diverse range of sympatric species that use its burrows for refuge and food resources. The bilby has experienced a severe decline over the last 200 years, and the species is now restricted to ~20% of its former distribution. Introduced predators, such as the feral cat (*Felis catus*), have contributed to this decline. We used camera traps to monitor bilby burrows at four sites in Western Australia, where bilbies were exposed to varying levels of cat predation threat. We investigated the impact of feral cats on bilby behavior at burrows, particularly during highly vulnerable periods when they dig and clear away soil or debris from the burrow entrance as they perform burrow maintenance. There was little evidence that bilbies avoided burrows that were visited by a feral cat; however, bilbies reduced the time spent performing burrow maintenance in the days following a cat visit (*P* = 0.010). We found the risk posed to bilbies varied over time, with twice the cat activity around full moon compared with dark nights. Given bilby burrows are an important resource in Australian ecosystems, predation by feral cats and the indirect impact of cats on bilby behavior may have substantial ecosystem function implications.

## INTRODUCTION

The relationship between predators and prey is an integral component of any ecosystem. Prey that fails to escape from a lethal predator will die, but even nonlethal injuries or nonconsumptive predator effects can result in reduced fitness (Peacor and Werner 2001; Hammill et al. 2015). Predator–prey interactions are, therefore, a major force driving evolutionary change in behavioral responses to either avoid or escape a predator (Cooper and Blumstein 2015). However, it is not only direct impacts of predators that can shape ecosystems. Even perceived predation risk influences prey species’ behavioral responses, including their willingness to undertake social activities, group size, and structure (e.g., Hunter and Skinner 1998), where and when they feed (e.g., Hochman and Kotler 2006), and when to resume feeding after disturbance (e.g., Lima and Bednekoff 1999). The perception of predation therefore has cascading impacts down food chains, influencing whole landscapes (Laundré et al. 2010).

Generally, moving prey are more likely to be seen by predators, and therefore increased predation risk can often result in reduced general activity levels (Lima and Bednekoff 1999). Some specific behaviors can also increase vulnerability. First, this may be because the behavior itself takes away time that can otherwise be spent being vigilant. For example, feeding can increase vulnerability, with animals needing to find an optimal balance between feeding and vigilance (Dall et al. 2001; Hochman and Kotler 2006), trading off their own food requirements with their safety risk. Social behavior such as allogrooming is also often balanced with predation risk (e.g., Blanchard et al. 2017). Second, some behavior further compromises the animal’s ability to perceive danger. For example, Rattenborg et al. (1999) found that a higher proportion of mallard ducks (*Anas platyrhynchos*) sleeping on the edge of a group (a position in the group that has a higher predation risk) used uni-hemispheric sleep, allowing them to remain vigilant, and oriented the open eye away from the group’s center compared to the birds sleeping in the center of the group. Digging and burrow maintenance are similarly likely to compromise an animal’s ability to perceive danger as the animal has its head in the burrow (blocking vision and hearing) while the act of digging itself creates noise that would attract attention as well as mask the sounds of an approaching predator (Rabin et al. 2006; Chan et al. 2010). Carrying out these specific behaviors may increase the vulnerability for prey species and therefore their predation risk, resulting in altered activity budgets in the presence of a threat.

As the risk of being preyed upon may vary greatly on a seasonal, daily, or even a minute-by-minute basis, perception of predation risk is likely to be influenced by temporal patterns in predator activity. Environmental factors such as lunar illumination influence nocturnal prey and predators’ activity patterns (Prugh and Golden 2014), and by extension how prey respond to predation risk (Griffin et al. 2005; Navarro-Castilla and Barja 2014). The predation risk allocation hypothesis (Lima and Bednekoff 1999) predicts that if predators were more successful with increasing lunar illumination, prey species would become lunar phobic and shift activity to less bright lunar phases where possible (Palmer et al. 2017). Another possibility is that prey may shift away from behavior that increases their vulnerability during risky time periods, decreasing their exposure by altering their activity budget.

Our study investigates the impacts of feral cats (*Felis catus*) on the burrowing behavior of the greater bilby (*Macrotis lagotis*). The bilby is a semi-fossorial, nocturnal mammal. The construction and maintenance of burrows by bilbies, during which they dig and clear away soil or debris from the burrow entrance, contributes to soil processes by increasing the heterogeneity of soil structure and increasing water infiltration, and such digging is important in shaping the ecology of Australian ecosystems (Fleming et al. 2014; Mallen-Cooper et al. 2019). Furthermore, their burrows provide shelter, foraging, and hunting opportunities for a variety of species, including birds, mammals, reptiles, and invertebrates (Hofstede and Dziminski 2017; Dawson et al. 2019). Bilbies therefore play an important role as ecosystem engineers (Jones et al. 1994). However, the bilby has experienced a severe decline in distribution and abundance due, in part, to predation from introduced predators such as the feral cat (Burbidge and McKenzie 1989; Woinarski et al. 2015). Feral cats target their hunting areas around areas of greater prey activity, and bilby burrows are foci for their attention (Moseby and McGregor 2022). Furthermore, many reintroductions of bilby populations are successful only in predator-free sanctuaries (Moseby and O’Donnell 2003; Berris et al. 2020), and in a translocated population exposed to cats, Ross et al. (2019) reported that all known fate bilby mortalities were consistent with cat predation.

We used burrow maintenance activity as a measure of bilbies’ perceived predation risk to feral cats. At a bilby population where feral cats were present, we used camera traps to compare bilby behavior at their burrows to quantify whether bilbies altered their (1) burrow use or burrow maintenance behavior after a visit by a feral cat, and (2) whether there was greater predation risk (likelihood of cat visitation to burrow) with moon phase. Comparing across four sites that had different levels of exposure to cats, we asked (3) whether bilby behavior differed by site in terms of the amount of time performing burrow maintenance between sites, or whether burrow maintenance was performed at different times of the night. Finally, we also asked a methodological question at one of the sites, (4) comparing whether scoring bilby behavior from photos was similar to scoring behavior from videos.

## METHODS

### Study sites

This study was conducted at four sites across Western Australia (Table 1). Feral cats were present at the study site in the West Kimberley region with an overall average of 1 cat detection on camera per 100 trap nights over the monitored period. The other three sites were free from terrestrial mammalian predators. Mt Gibson Wildlife Sanctuary, located ~375 km north-east of Perth, was the largest fenced population studied, with a 7832-ha sanctuary surrounded by a feral predator-proof fence. Barna Mia Native Animal Sanctuary, located ~150 km south-east of Perth, has bilbies housed within two 4-ha feral predator-proof enclosures. The captive population at Kanyana Wildlife Rehabilitation Centre, located approximately 30 km east of Perth, housed each bilby in a small enclosure (~15 m^2^) on sandy substrate (with a wire mesh floor) with an artificial “burrow” that consists of a wooden “nest” box with a PVC pipe leading out to the entrance.

**Table 1:** Details of study sites in Western Australia, including research partners, presence or absence of predators, the number of cameras deployed at bilby burrows, and the period each site was monitored for

Study sites, research partners	Predators present:	Cameras (number and Reconyx model(s))	Time deployed (total trap nights)
West Kimberley region^a^, 18°000 S, 123°000 E	Y	127 (HC500, HC600, PC900, UXR6)	Jun 2014 – Sep 2016 (5414 trap nights)
Fenced populations:			
Mt Gibson Wildlife Sanctuary, AWC 29°630 S, 117°236 E	N	17 ^b^ (HF2X)	June – Aug 2021 (582 trap nights)
Barna Mia Native Animal Sanctuary, DBCA 32°462 S, 116°553 E	N	17 (HF2X)	May – June 2021 (765 trap nights)
Captive population:			
Kanyana Wildlife Rehabilitation Centre 32°019 S, 116°040	N	8 (HF2X)	June 2021 (10 trap nights)

AWC, Australian Wildlife Conservancy; DBCA, Department of Biodiversity, Conservations and Attractions.

^a^Camera trap data collected as part of a monitoring project (Dawson et al. 2019).

^b^Two cameras were each deployed at eight bilby burrows, with one burrow having one camera.

### Camera traps

At each site, the area was exhaustively searched for burrows in a systematic manner, with cameras placed on as many burrows as could be found, while eight enclosures were chosen at Kanyana based on the ease with which the opening of the artificial burrow could be viewed by the camera. Cameras were mounted on a metal stake (or the enclosure mesh for captive animals) at a height of 0.5 m, at a distance of 0.5–1.5 m from the burrow opening (distance varying depending on the surrounding vegetation). Cameras were aimed oblique to the burrow entrance to ensure animals would pass across the field of detection (rather than move toward it), maximizing detection probability (Meek et al. 2014).

“Still” camera traps were set to passive infra-red (PIR) trigger, five rapid-fire images, no quiet period, high sensitivity, with an infrared flash. At Mt Gibson, each bilby burrow had a pair of cameras: one “still” camera trap and a second “video” camera trap set to PIR trigger, high sensitivity, and programmed to take a 15-s video per trigger.

### Image and video analysis

**Figure 1:**
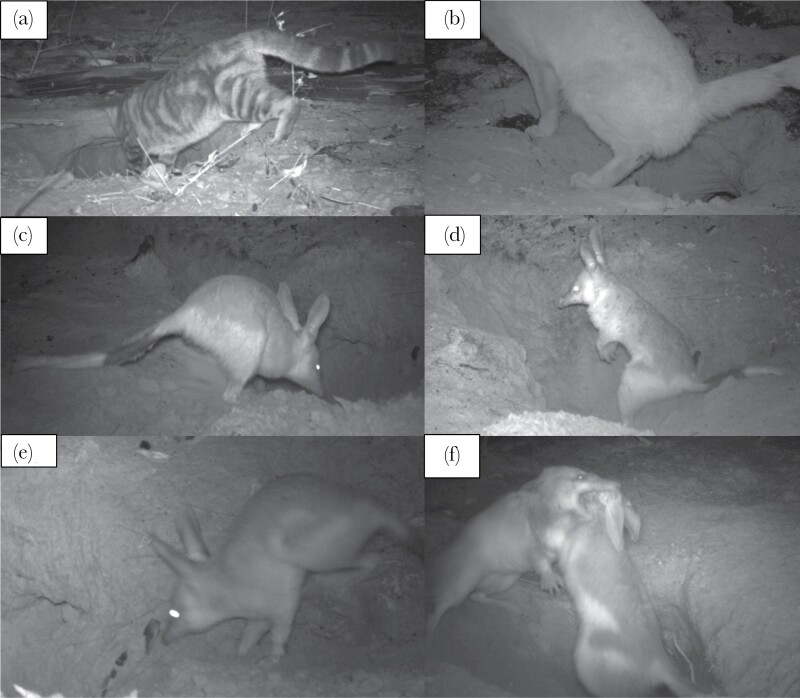
Example photos showcasing feral cat (*Felis catus*) and greater bilby (*Macrotis* lagotis) behavior categories scored in the present study. (a) Cat investigating a bilby burrow, and (b) a cat urinating at the entrance of a bilby burrow. Bilby behavior included (c) Maintenance: actively digging or clearing away soil or debris from burrow entrance, (d) Vigilance: interrupting activity to stand (bipedal or on all four legs) immobile (as evident across a series of images) with head and ears erect, (e) Interacting: all other actions and behaviors (animal moved across a series of images) including entry into burrow, exit out of burrow and social interactions as shown in (f) (bilby brawl).

**Figure 2:**
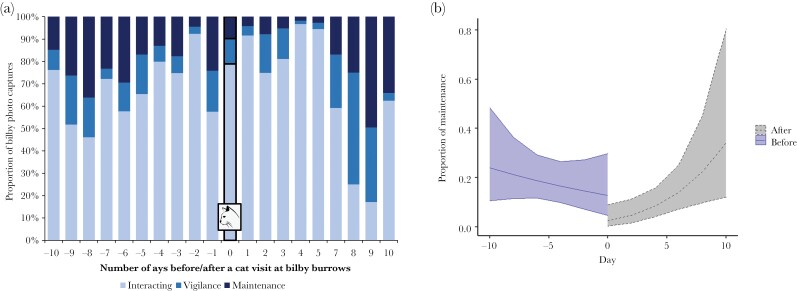
(a) Overall behavioral profile of bilbies across 24 monitored bilby burrows (across five populations in the West Kimberley, Western Australia), showing the 10 days before and 10 days after a visit by a feral cat (designated as Day 0; highlighted with the bold outline). Each bar shows the average proportion of images showing “Interacting,” “Vigilance,” and “Maintenance” behavior on each day. There is no data for the sixth day as none of the bilbies returned to these monitored burrows on that day. (b) Model showing mean (±95% CI) burrow maintenance as a proportion of bilby photo captures 10 days prior to and 10 days following a visit by a feral cat. Values were derived from the regression model, using the “ggeffects” package with all other continuous variables held at fixed median levels and categorical values at the most frequent category.

Photos and videos of animals were identified to species or genus level and tagged in the metadata using the application “digiKam” (digikam team 2001–2021). The metadata from the images was extracted using the package “camtrapR” (Niedballa et al. 2016). We counted up the total number of species observed interacting with bilby burrows (“burrow commensals” as defined by Dawson et al. 2019). Any photos of a cat captured on the burrow cameras was defined as a cat visit, and included 6 visits during daytime and 56 visits during night time. No attempt was made to identify individual animals, but all bilby images in this study were further categorized based on the action of the animal as follows:

(1) “Maintenance,” where the bilby was actively digging soil away from the burrow entrance or clearing away debris such as branches (Figure 1a);
(2) “Vigilance,” where the bilby interrupted their activity to stand immobile, bi-pedal or on all fours with their head and ears erect (Figure 1b); or
(3) “Interacting,” included all other behaviors such as entering or exiting the burrow, passing by the burrow, and inspection of the burrow (Figure 1c,d).

We used the proportion of images for each burrow for each night to quantify the proportion of time spent exhibiting a particular behavior. Videos were scored for these same categories (as above) as state events using “Behavioral Observation Research Interactive Software” (BORIS) (Friard et al. 2016). The time budget function in BORIS was used to calculate the proportion of time spent on a particular behavior.

### Statistical analyses

All data analyses were conducted in R (R Core Team 2021).

The first two experimental questions, comparing (1) bilby burrow use or burrow maintenance behavior after a visit by a feral cat, and (2) whether there was greater predation risk (likelihood of cat visitations at burrows) with moon phase, included only “active” burrows (24 burrows where both bilbies and cats were recorded at least once during the monitored period) across five populations in the West Kimberley (the only study site with cat activity). We compared the 10 days before and the 10 days after a feral cat visit to maximize our ability to detect a response to the cat visit. This period was not based on any precedent, but rather on a post-hoc assessment of the frequency of feral cat detections within the dataset. The monitoring period was truncated if the camera had been deployed less than 10 days before a cat visit, or was removed within 10 days of recording a cat. We considered cat visits to be independent of each other if they were separated by more than 21 days from other cat visits, but for instances where consecutive cat visits to a burrow were within a shorter time frame, only data for the first cat visit was used and the “after” period was truncated. The average duration of monitoring was therefore 8.59 ± 2.74 days “before” a cat visit and 9.21 ± 2.40 days “after” a cat visit. Differences in monitoring duration were accounted for by calculating the detection rate (number of events divided by the number of days that the camera was active, i.e., “trap nights”).

### Do bilbies alter their burrow use or burrow maintenance behavior after a visit by a feral cat?

To test for the effect of cat visits on bilby burrow use, we fitted Generalized Linear Models (GLMs) using two dependent variables: (1) bilby presence (the number of days a bilby was present as a ratio of the number of days of monitoring for that burrow), and (2) bilby activity (camera detection rate). The predictor variable was cat exposure (before or after a cat visit), where each row of the dataset was of an independent cat visit, that was scored “0” if before the cat visit, or “1” if after the cat visit. We evaluated model fit using the quartile-quartile plot function in the “DHARMa” package (Hartig 2021), which indicated an overdispersion of residuals due to the high proportion of zeroes. Given that our data consisted of continuous values and were highly zero-inflated, the GLMs were fitted with a Tweedie link using the “tweedie” package (Dunn and Smyth 2008) in R, where the variable power was specified to maximize model fit.

To test for the impact of cat exposure on bilby burrow maintenance behavior, we fitted a GLM with a Tweedie link to the proportion of bilby camera trap photos classified as burrow maintenance as the dependent variable, with cat exposure (before or after a cat visit), day of monitoring (10 days before and 10 days after a cat visit), and the interaction term of cat exposure and day as predictor variables. The tweedie package does not allow for the inclusion of random factors (to address potential pseudoreplication due to multiple data points collected from the same burrows); however, the data had minimal risk of pseudoreplication, with two-thirds of the burrows analyzed only having a single cat visit that met our criteria for inclusion, while only 7 out of 22 burrows analyzed had two cat visits that met our criteria for inclusion. The proportion of images where bilbies were performing burrow maintenance (expressed as a proportion of the total of bilby detections) was calculated for each day for the 10 days before and the 10 days after each independent cat visit. Relationships were plotted using the “ggeffects” package (Lüdecke 2018).

### Do bilbies face greater predation risk with moon phase?

Cat activity (independent detections per 100 trap nights) was calculated on a given night that the camera was open across the 24 bilby-active burrows monitored in the West Kimberley. To test if cat activity at bilby burrows was influenced by the moon phase, we fitted a GLM with a Tweedie link to the number of independent cat detections as the dependent variable, with lunar illumination (0 as a new moon, and 1 as a full moon extracted of each day of monitoring using the “lunar” package; Lazaridis 2014), bilby activity (number of detections per 100 trap nights), and the interaction of lunar illumination and bilby activity as predictor variables.

### Does bilby burrow maintenance behavior differ between sites?

To test if bilbies spent a different proportion of their time performing burrow maintenance across the four sites, we fitted a GLM with a Tweedie link to the proportion of bilby images showing burrow maintenance at each burrow as the dependent factor, with site (West Kimberley, Mt Gibson, Barna Mia, Kanyana) as the predictor variable. We then compared the means of the proportion of burrow maintenance for each site using a Tukey analysis using the “emmeans” package (Lenth et al. 2018). To test if bilbies performed burrow maintenance at different times of the night across the four sites, we fitted non-parametric kernel density curves using the “overlap” package (Ridout and Linkie 2009). If required, we minimized duplicates by altering identical timestamps by 0.00001 s in the raw data.

### Is scoring bilby behavior from photos similar to scoring bilby behavior from videos?

Footage from Mt Gibson was used to test if scoring bilby behavior from photos was as consistent as scoring behavior from continuous footage collected through video cameras. Because there is published evidence of observer bias influencing the interpretation of camera trap and video footage (Foster and Harmsen 2012; Cruickshank and Schmidt 2017), video and image analysis was performed by the same observer. Videos were collected from eight burrows. Five nights with the highest bilby activity were selected from each of the eight burrows, where activity ranged from 3 to 156 videos per night. Bilby behavior observed from videos was scored using the same categories as photos. The proportion of each behavioral category per night for videos were derived using the “Time Budget” function in BORIS. To test if there was a significant correlation in the activity budget derived from photos or videos, we fitted a Generalized Linear Mixed Model to the proportion of each behavioral category from video cameras (as a proportion of all the footage collected on each night) as the dependent variable, with the proportion from photos (proportion of the respective behavioral categories derived from analysis of photos on the corresponding night) as the predictor variable, and burrow ID as a random factor.

We also quantified the logistical constraints of collecting videos from camera traps compared to photos, quantifying the average file space on SD cards and battery life from 16 “video” and “photo” camera pairs. We used the “survival” package (Therneau 2021) to create a survival plot showing the remaining number of active cameras over time.

## RESULTS

In the West Kimberley, 127 burrows were monitored for a total of 5414 trap nights, during which time 74 of the monitored burrows were active (at least one bilby detection), resulting in a total of 21 426 bilby photos. Cats (total of 74 photos) were detected at 24 of the 74 bilby-active burrows. At Mt Gibson, there were nine monitored burrows, for a total of 582 trap nights, with 23 972 bilby photos. At Barna Mia, all 17 monitored burrows were active, for a total 748 trap nights, with a total of 1264 bilby photos. At Kanyana, there were eight monitored bilbies in separate enclosures, for a total of nine trap nights, with a total of 7491 bilby photos.

### Do bilbies alter their burrow use or burrow maintenance behavior after a visit by a feral cat?

Cats observed at the bilby burrows displayed a range of behaviors from moving past the burrow with no apparent interest, to investigative behavior (such as sniffing, and inserting their head in the burrow entrance). At least one cat was observed urinating at the entrance of the bilby burrow (Figure 1b). There was no evidence that bilbies altered their frequency of use after a visit to the burrow by a feral cat for 24 active bilby burrows in the West Kimberley that also recorded at least one cat (distributed across five separate populations, and monitored for total of 2433 trap nights). There was no significant effect of cat exposure on bilby presence (*P* = 0.200, Table 2a–i), or bilby detection rate (*P* = 0.429, Table 2a–ii). However, bilbies significantly altered their time budget around burrows after exposure to a feral cat. Before a cat visit, bilbies spent an average 15% of their time in front of the camera performing burrow maintenance (Figure 2a). By contrast, after a cat visit, the average dropped to 5% for the first five days, before increasing again seven days after the cat visit (Figure 2a). There was a significant difference in the overall proportion of bilby burrow maintenance with cat exposure (*P* = 0.050, Table 2b). The proportion of burrow maintenance also showed a significant interaction between cat exposure and day of monitoring (*P* = 0.010, Table 2b) capturing the gradual return to burrow maintenance after a cat visit (Figure 2b).

**Table 2:** Results of GLMs with a Tweedie link testing for the impact of feral cat exposure (10 days before vs. 10 days after a known feral cat visit) on (a) bilby burrow use and detection rate, or (b) bilby burrow maintenance behavior across five populations in the West Kimberley, Western Australia. Also shown is (c) the relationship between bilby activity (detections per 100 trap nights) with lunar illumination, and (d) the relationship between cat activity (detections per 100 trap night) with lunar illumination and bilby detection at 24 monitored burrows that recorded both at least one cat and one bilby

	Estimate	SE	*t*-value	*P*-value
Data for burrows visited by cats (10 days before vs. 10 days after a known feral cat visit).				
(a) Bilby burrow use				
(i) Bilby presence (proportion of days monitoring)	0.033	0.026	1.30	0.200
(ii) Bilby detection rate (per 100 trap nights)	−0.283	0.355	−0.797	0.429
(b) Bilby burrow maintenance behavior				
Cat exposure (before/after feral cat visit)	0.185	0.093	6.790	**0.050**
Day of monitoring	0.033	0.012	2.665	**0.009**
Cat exposure × Day of monitoring	−0.042	0.016	−2.640	**0.010**
Data for each day of survey for 24 monitored burrows that recorded both at least one cat and one bilby				
(c) Bilby activity				
Lunar illumination	−0.196	0.208	−0.944	0.346
(d) Cat activity				
Lunar illumination	1.660	0.642	2.586	**0.010**
Bilby detection rate	0.007	0.008	0.908	0.365
Lunar illumination × Bilby detection rate	0.030	0.016	1.899	0.059

Bold values indicate either a significant *P*-value.

### Do bilbies face greater predation risk with moon phase?

**Figure 3:**
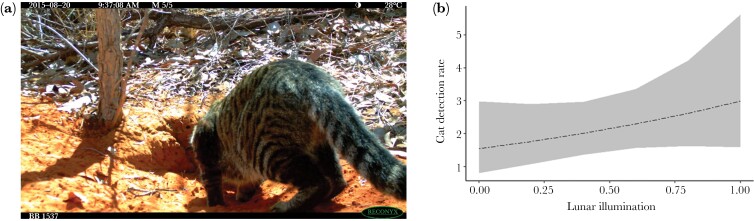
(a) Feral cat (*Felis catus*) investigating a bilby burrow in the West Kimberley. (b) Model showing the predicted mean (±95% CI) relationship between lunar illumination and cat activity (detection rate per 100 trap nights) at 24 bilby burrows across five populations in the West Kimberley, accounting for the interaction between lunar illumination and bilby detection rate (not shown). Values were derived from the regression model using the “ggeffects” package with all other continuous variables were held at fixed median levels and categorical values at the most frequent category.

There were 59 independent cat visits recorded across five West Kimberley populations. Cat activity at bilby burrows was significantly positively correlated with lunar illumination (*P* = 0.010, Table 2d; Figure 3b), with 1.93 times more cat detections at bilby burrows for full moonlit nights compared with new moon nights. This relationship indicates substantially greater predation threat for bilbies on full moon nights, although bilby detection rate was not influenced by lunar illumination (*P* = 0.346, Table 2c). There was no significant relationship between cat detection rate and bilby detection rate (*P* = 0.365, Table 2d), indicating that cat activity and bilby activity at the same burrows were not correlated, although there was a suggestion of an interaction between lunar illumination and bilby detection rate (*P* = 0.059, Table 2d).

### Does bilby burrow maintenance behavior differ between sites?

**Figure 4:**
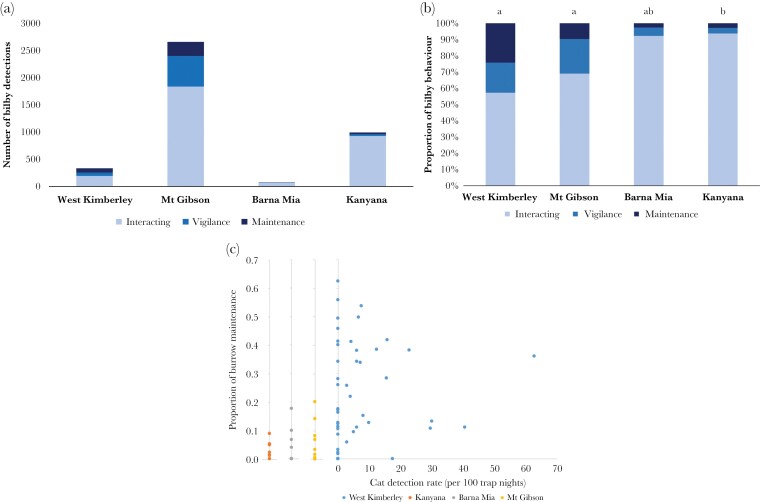
Overall behavioral profile of bilbies across four sites expressed (a) as number of events per monitored burrows, and (b) as a proportion of all events. Each bar shows the proportion of images classified as “Maintenance,” “Vigilance,” and “Interacting” at each of the four sites in this study: seven wild populations in the West Kimberley (74 burrows), fenced populations at Mt Gibson (9 burrows) and Barna Mia (17 burrows), and a captive population at Kanyana (8 burrows). Letters represent significant differences in the mean proportions of events showing bilby burrow maintenance between sites, according to Tukey’s HSD test. (c) Proportion of bilby burrow maintenance behavior at each of the 108 bilby-active burrows across the four sites; only the West Kimberley populations had feral cats present.

**Figure 5:**
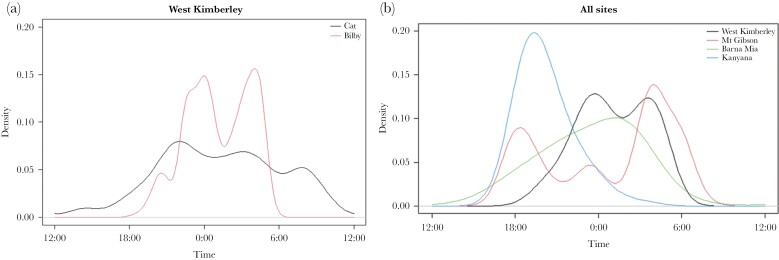
(a) Temporal activity of all cat activity at bilby burrows compared with bilby burrow maintenance behavior at the West Kimberley site only. (b) Temporal activity of bilbies’ burrow maintenance behavior across four sites; seven wild populations in the West Kimberley (74 burrows), fenced populations in Mt Gibson (9 burrows) and Barna Mia (17 burrows), and a captive population in Kanyana (8 burrows).

A total of 108 bilby-active burrows were monitored across the four study sites, with a total of 7918 camera trap nights. Overall, bilbies in the West Kimberley spent a greater proportion of their time performing burrow maintenance, while bilbies at Barna Mia and the captive animals at Kanyana spent a smaller proportion of their time conducting burrow maintenance (*P* = 0.001 for pairwise comparisons, Figures 4 and 5). Dawson et al. (2019) identified 45 taxa that actively interacted with bilby burrows at the West Kimberley. Raw species richness at Mt Gibson and Barna Mia shows that there were 16 and nine species (respectively) that interacted with bilby burrows, while the animals at Kanyana were housed individually (i.e., no commensals).

The temporal activity of bilbies and cats in the West Kimberley had a 61.1% overlap in circadian timing of photo captures, with the activity peaks of both species being significantly different (Watson-wheeler value: 127.55, *P* = <0.001. The temporal activity of bilbies in the West Kimberley and Barna Mia were the most similar out of the other sites with 71.1% overlap, and both sites showing similar activity peaks just past midnight (Watson-wheeler value: 0.353, *P* = 0.838). The temporal activity of bilbies in the West Kimberley and the captive population at Kanyana were the most distinct from each other, overlapping by only 29.7% and showing significantly different activity peaks (Watson-wheeler value: 311.8, *P* = <0.001), where the captive bilbies (Kanyana) performed the majority of their burrow maintenance at the start of the night around dusk (~18:00 h). Bilbies at Mt Gibson were primarily crepuscular in terms of their burrow maintenance activity.

### Is scoring bilby behavior from photos similar to scoring behavior from videos?

**Figure 6:**
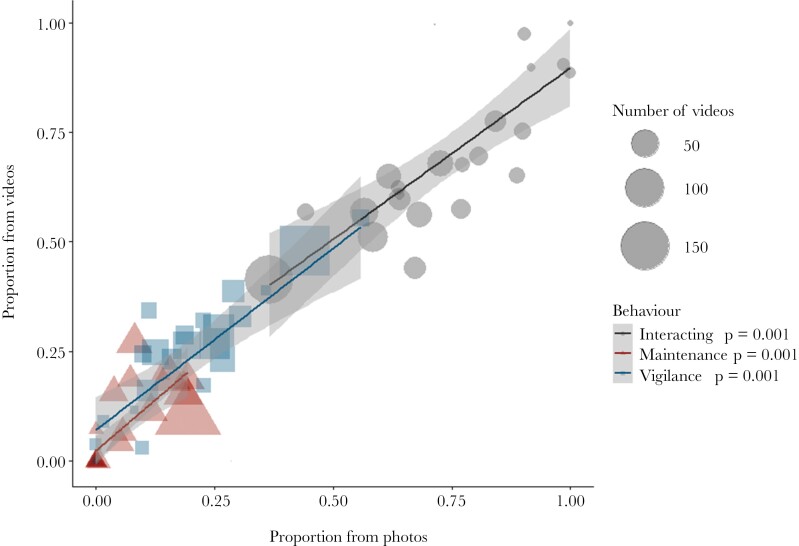
Model showing the relationship between the proportion of each behavioral category derived from photos or from videos at 8 burrows in Mt Gibson recorded across 39 nights. Each data point is the bilby activity on a given night at one burrow. Videos for each night range from 3 to 156. “Interacting” shown in black circles; “Maintenance” shown in red triangles; “Vigilance” shown in blue squares..

A total of 6492 images and 3.55 h of video of bilbies (for 8 burrows in Mt Gibson collected over 38 nights) were analyzed to compare the behavior time budgets derived from photo and video analyses. There was a significant correlation for the proportions of each behavior category estimated from the photos and videos (*P* = 0.001 for each behavior, Figure 6). In terms of logistic comparison between data collected from photos or videos, on average, video cameras took up approximately twice the file space (4.57 GB) of a “still” camera (2.65 GB) and twice as many video cameras (9) had depleted batteries compared to “still” cameras (4) by the end of the 38-night study period.

## DISCUSSION

Contrary to our predictions, bilbies did not avoid burrows that were visited by a feral cat. However, bilbies did alter their behavior around these burrows, decreasing the proportion of time spent performing burrow maintenance for ~5 days after a visit by a feral cat. Our results support the assumption that burrow maintenance is a risky activity that can increase vulnerability by attracting the attention of potential predators or masking the sound of their approach (i.e., distracted prey hypothesis; Chan et al. 2010). This result is consistent with predictions under the predation risk allocation hypothesis (Lima and Bednekoff 1999) that animals should minimize risky behavior in the presence of a potential predation threat. This result suggests that burrow maintenance is balanced with foraging and vigilance behaviors according to the bilby's environment and, critically, their exposure to predation risk.

### Do bilbies alter their burrow use or burrow maintenance behavior after a visit by a feral cat?

The lack of burrow avoidance in response to cat presence may be due to multiple reasons. First, intact bilby burrows may be too valuable to abandon. Burrows are highly important as a refuge against predators. For example, Mojave Desert tortoises (*Gopherus gassizzi*) increased refuge-seeking behavior when they encountered cues of their principal predator, the coyote (*Canis latrans*) (Nafus et al. 2017). Burrows represent a significant investment in time and effort spent digging and are therefore not readily replaced, and consequently a burrow is not likely to be abandoned if perceived risk is insufficient to deter the animal from continuing to use it, or there is some (low level) perceived risk at all burrows. In contrast with our study, for a fenced sanctuary without cats, Moseby et al. (2012) found that “trained” bilbies (which were hand-captured and handled with cat scent equipment and a cat carcass) were more likely to move away from burrows treated with cat scent compared to “untrained” bilbies (with no prior cat exposure). Steindler et al. (2018) similarly showed that bilbies altered their behavior around burrows in the presence of dingo scat, with bilbies less likely to be photographed fully emerged at the burrow entrance (noting that they showed no difference in bilby behavior between cat and rabbit scats or a procedural control). Ross et al. (2019) found that cat-exposed bilbies (from a population that had been living with cats for 2 years) placed into a pen with an artificial burrow were warier than predator-naive bilbies, spending less time moving and more time in cover. Van der Weyde et al. (2022) also found that quoll-exposed bilbies were generally more wary and neophobic compared to quoll-naive bilbies. In our study in the West Kimberley, the bilbies are likely to encounter cats or signs of cats (feces, scent marks) both at the burrow and away from it as they move between refuge and foraging places. While we had no evidence that bilbies avoided burrows that had been visited by cats, we showed a significant change in their behavior around the burrows, spending less time in burrow maintenance for a period of ~5 days after the cat visit. The data show a range of different bilby behavioral responses to cat presence, suggesting that they are likely to modify their behavior around burrows as well as in other parts of the landscape generally.

Second, our interpretation that bilbies avoided using burrows that had been visited by a feral cat is limited by the camera trapping methodology used. We were unable to determine if bilbies resided in the same burrow as they were last detected during the day, as the cameras were unable to reliably detect bilbies entering or exiting the burrows. Additionally, our cat detections only included known cat visits to monitored burrows and was limited to activity immediately around the entrance of the burrows. Our cat detection rates, therefore, do not reflect the actual cat density or activity in the West Kimberley.

### Do bilbies face greater predation risk with moon phase?

Feral cats were twice as active around bilby burrows on full moonlit nights compared to new moon nights. If predation risk increases with lunar illumination, the predation risk allocation hypothesis (Lima and Bednekoff 1999) predicts that prey species would decrease their activity during the full moon (Griffin et al. 2005; Prugh and Golden 2014). A number of studies have examined the impact of lunar illumination on prey species supporting predictions of thepredation risk allocation hypothesis; for example, Linley et al. (2021) found that native prey species decreased their activity with increasing lunar illumination. However, there has been surprisingly few studies that have examined the impact of lunar illumination on cat hunting behavior. Moseby and McGregor (2022) found that cats spent longer around prominent prey cues such as bilby burrows and signs of bilbies, although they did not investigate the effect of lunar phase. Gilmore (2016) examined the impact of lunar illumination on the hunting behavior of a number of introduced mammalian predators in New Zealand, but found no effect of moonlight for cat activity. Given our finding that cats are more active on moonlit nights, we expected bilbies to hide in their burrows and therefore, not be seen around the entrance. However, bilbies did not appear to respond to the apparent increase in predation risk as bilby detection rate was not significantly affected by lunar illumination.

### Does bilby burrow maintenance behavior differ between sites?

Bilbies showed differing time budgets between sites where cats were present and feral predator-free sites, although there was no direct relationship with the amount of feral cat activity. We predicted that bilbies in sites with higher predation risk, such as those in the West Kimberley, would spend less time performing burrow maintenance to reduce their vulnerability in the presence of predators compared to sites with no cat presence. Contrary to this prediction, the West Kimberley bilbies performed more burrow maintenance compared to the fenced or captive populations. Factors other than predation threat could potentially explain this phenomenon.

First, environmental factors such as temperature, rainfall, and soil type may influence the amount of digging required across the different study sites. For example, hotter temperatures may result in deeper burrows to maintain cooler temperatures and stability (Chapman 2013), as bilbies are prone to heat stress due to their inability to sweat (Johnson 1989), which in turn may lead to greater levels of burrow maintenance for deeper burrows. Soil type is also expected to influence the amount of digging required, particularly the soil components that influence its adhesive properties. We were unable to account for temperature, rainfall, or the effect of soil type (apart from noting that bilbies preferred sandy areas) in the present study, however, due to the small number of study sites that do not capture variability in these measures.

Second, burrow maintenance could be influenced by the number and type of burrow commensals. Lastly, male and female bilbies are also likely to perform different amounts of burrow maintenance. For example, females have smaller home ranges than males (Moseby and O’Donnell 2003; Berris et al. 2021) and may spend more time maintaining fewer burrows, particularly when they are caring for young and must remain at a maternal burrow over a longer period.

The differing temporal profiles at each site suggest bilbies at sites without cats (e.g., Mt Gibson bilbies) perform burrow maintenance at a time when cats would be most active. Additionally, the bilbies’ varying temporal profiles at each site may also be influenced by food availability, and perhaps environmental factors such as temperature and rainfall. Burrow maintenance is an energy-costly behavior, and we hypothesize that perhaps in areas of low to moderate food availability, bilbies cannot afford to expend their energy in burrow maintenance before foraging. For example, the captive bilbies in Kanyana performed the majority of their burrow maintenance in the first few hours of the night. Food is readily available to them, and they did not need to forage as much as wild animals would. In the West Kimberley and Barna Mia, peak burrow maintenance occurred in the middle of the night, possibly after the animal had returned from foraging. Even in Mt Gibson, where burrow maintenance activity was crepuscular, the majority of maintenance occurred toward the end of the night, when the animal would have returned to the burrow after foraging. Second, external factors such as weather conditions could also impact the time of night that burrow maintenance is performed. Particularly, temperature may influence when bilbies emerge from their burrows; Jones (1924) notes that bilbies reduced activity in cold weather, and Gibson and Hume (2004) suggest that bilbies may avoid low temperatures to minimize thermoregulatory costs with bilbies finding different conditions to be advantageous for foraging.

### Is scoring bilby behavior from photos similar to scoring behavior from videos?

We found that scoring behavior from photos and videos were strongly correlated, which supports the analysis of behavior from photo images. We had expected scoring of “Vigilance” to be underestimated for photo captures as vigilance behavior generally minimizes movement of the animal, therefore the likelihood of continually triggering the camera, and could also be scored simply as “interacting” if it was taken out of context, although the strong correlation indicated that this was not the case.

Videos arguably allow better detection of the nuances in behavior, although the observer experienced the onset of fatigue in a shorter period when scoring videos compared to scoring photos. Photos were not scored in isolation, and if possible, the five images in sequence (from each trigger) were viewed together, allowing for some interpretation of behavioral context. As a result, it was less time consuming and efficient to score thousands of photos spanning multiple nights, than hundreds of videos that were recorded on a single night. For logistical reasons of file size and battery life, scoring from photos was also preferable. There are both advantages and disadvantages to using either videos or photos to score behavior, and the choice to use either (or both) is dependent on research goals and the type of data and data analysis methods that are desired.

### Limitations

Our conclusions about cat presence were based on single photos and cat detection rate from camera captures at bilby burrows. This may have resulted in an overestimation of cat activity because we did not distinguish between individuals. We were also not able to detect or estimate the actual density and activity of feral cats in the West Kimberley.

Recording behavior from camera trap images was also problematic for the captive population at Kanyana, where the small enclosure meant that the camera’s field of vision consisted of half the enclosure. As a consequence, the cameras were constantly triggered, even when the bilby was away from the entrance of the burrow, draining the batteries within one or two nights. However, we do not believe that having the cameras open for a longer period would have changed the recorded behavioral profile of Kanyana bilbies, as the behavior of these captive animals was unlikely to change significantly.

Finally, our time budget analyses were based on the proportion of time that bilbies spent carrying out burrow maintenance and not the total amount of time spent on this activity. The marked differences in activity between burrows precluded a meaningful comparison of absolute time spent carrying out burrow maintenance.

## CONCLUSIONS

We used burrow maintenance as a novel method to measure the bilbies’ perceived predation risk to feral cats and showed that although the visit of a cat did not appear to change bilbies’ burrow use, even their presence altered bilbies’ behavioral time budget for up to 5 days after its visit. Bilbies in the West Kimberley are also potentially more vulnerable with increasing lunar illumination when there are more cat detections at burrows, demonstrating context dependence to this predation risk. As bilbies are an ecosystem engineer, their digging activity (even as they forage) influences multiple ecosystem processes such as soil properties, plant germination rates, and their burrows also provide refuge for other burrow commensals. Therefore, our study has shown that cats reduce bilbies’ digging activity after a visit, and this behavioral change not only has implications for bilbies but also for soil, flora, and other fauna species in areas where cats are present.

## Data Availability

Analyses reported in this article can be reproduced using the data provided by Chen et al. (2023).
